# Fulminant Influenza A Myocarditis Complicated by Transient Ventricular Wall Thickening and Cardiac Tamponade

**DOI:** 10.3390/idr14040065

**Published:** 2022-08-15

**Authors:** Milan Radovanovic, Igor Dumic, Charles W. Nordstrom, Richard D. Hanna

**Affiliations:** 1 Mayo Clinic Alix School of Medicine, Rochester, MN 55905, USA; dumic.igor@mayo.edu (I.D.); nordstrom.cw@mayo.edu (C.W.N.); hanna.richard@mayo.edu (R.D.H.); 2 Department of Hospital Medicine, Mayo Clinic Health System, Eau Claire, WI 54703, USA; 3 Department of Cardiology, Mayo Clinic Health System, Eau Claire, WI 54703, USA

**Keywords:** influenza A, fulminant myocarditis, myocardial edema, cardiac tamponade

## Abstract

Myocarditis is an infrequent complication of influenza infection that is most often diagnosed clinically in the setting of confirmed influenza infection and elevated cardiac enzymes. Pericarditis can also occur in cases of influenza myocarditis and may require pericardiocentesis for tamponade. Patients with fulminant myocarditis have cardiogenic shock; however, echocardiographic findings may be subtle, showing a preserved ejection fraction and diffuse left ventricular wall thickening (compared to baseline) due to inflammatory edema. Recognizing these echocardiographic findings in the appropriate clinical setting facilitates the early recognition of fulminant myocarditis. Therefore, we report a case of fulminant influenza A myocarditis in healthy 37-year-old women complicated by transient left ventricular wall thickening and tamponade, highlighting the importance of early diagnosis and supportive management for a successful outcome.

## 1. Introduction

Influenza viruses belong to the *Orthomyxoviridae* family and can be classified into A, B, and C types [1]. Based on the antigenicity of the surface proteins, they can be further subdivided into a combination of 16 Haemagglutinin (H1–H16) and 9 neuraminidase (N1–N9) subtypes [1]. While seasonal epidemics are common with influenza A and B, it has never been encountered with influenza C, which typically causes a mild upper-respiratory illness in young children [2]. Influenza viruses are responsible for significant morbidity and mortality mostly due to respiratory illnesses and place a substantial burden on healthcare systems around the world [1]. While cardiac-related complications, such as myocarditis, have been reported in approximately 0.4–13% of hospitalized patients with an acute influenza infection, pericarditis alone or coupled with myocarditis (i.e., myopericarditis) is quite rare and infrequently reported [3,4]. Acute myocarditis and pericarditis commonly coexist in clinical practice, which is not surprising given the common etiologic agent-cardiotropic viruses [5]. Pericardial involvement can occur in the acute phase of infection but can also manifest as a post-viral syndrome [1]. In contrast, echocardiographic findings of ventricular dysfunction and wall thickening resembling myocardial hypertrophy are usually present during the acute phase of infection due to myocardial edema and inflammation [6].

By reporting this case, we would like to raise awareness of these rare but fulminant extrapulmonary complications of influenza A infection—highlighting the importance of early diagnosis and supportive management for a successful outcome.

## 2. Case Presentation

A healthy 37-year-old Caucasian woman who was unvaccinated against seasonal influenza, and without previous cardiovascular history, presented to the emergency department (ED) with a 3-day history of progressively worsening dyspnea, orthopnea, and pleuritic retrosternal chest pain. One week prior to the onset of her presenting symptoms, she developed a viral prodrome with upper-respiratory symptoms consisting of chills, diaphoresis, myalgias, arthralgias, sinus congestion, pharyngitis, and a non-productive cough. Upon presentation to ED, the patient was found to be afebrile and normotensive, but tachycardic with a heart rate of 140 beats/minute, and tachypneic with a respiratory rate of 22 breaths/minute. Her body mass index (BMI) was 34 kg/m^2^ consistent with class I obesity. There was no elevated jugular venous pressure (JVP), nor pericardial friction rub on initial examination. No track marks or signs of intravenous (IV) drug use were noted on skin examination. An electrocardiogram (ECG) revealed sinus tachycardia with low QRS complexes voltage but without electrical alternans. The patient tested positive for influenza A by the nasopharyngeal real-time reverse transcriptase (RT)-PCR test. Initial laboratory workup demonstrated a leukocytosis of 16,700 cells per microliter, with neutrophilic predominance (85%), hemoconcentration with hemoglobin of 21.3 g/deciliter and hematocrit of 64.3%, and a platelet count of 415,000 per microliter. The chemistry profile was within normal limits, including intact kidney and liver functions. Inflammatory markers were elevated with C-reactive protein (CRP) and erythrocyte sedimentation rate (ESR) being 61.4 milligrams/liter and 120 mm/h, respectively. D-dimer was 0.86 micrograms/milliliter (reference value: 0.5 micrograms/milliliter). High sensitivity troponin-T was elevated at the 0.06 nanograms/milliliter (reference value 0.01 nanograms/milliliter), and N-terminal pro-Brain Natriuretic Peptide (NT-proBNP) was 1208 picograms/milliliter (reference value: less than 100 picograms/milliliter). Serial troponin measurements along with other pertinent labs throughout the hospital stay are displayed in Table 1.

**Figure 1:**
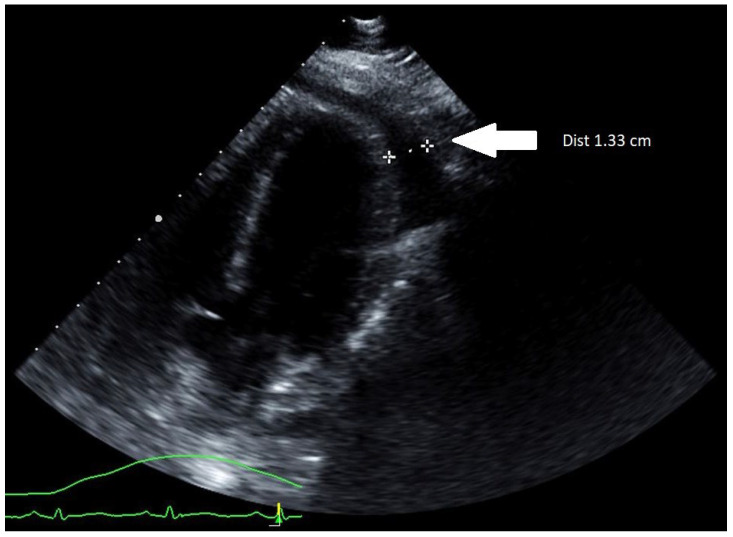
TTE (parasternal long-axis view) showing an increasing pericardial effusion measuring 1.33 cm (marked by two white stars).

Initial computerized tomography (CT) scan of the chest along with a transthoracic echocardiogram (TTE) showed a small pericardial effusion with normal ventricular size and function. The patient was started on systemic steroid therapy with methylprednisolone 40 milligrams IV every 6 h, colchicine 0.6 milligrams two times daily, along with Oseltamivir 75 milligrams two times daily for influenza A pericarditis. Over the following 12 h, the patient received 4 L of IV crystalloid saline without improvement in her heart rate. Subsequently, she developed hypotension with the median arterial pressure (MAP) falling below 55 mm of mercury leading to left radial arterial line placement. Upon connection of the arterial line to the pressure transducer, the arterial waveform demonstrated pulsus alternans and, at times, pulsus paradoxus. At this point, an urgent TTE was performed and demonstrated an increasing pericardial effusion now measuring 1.33 cm with dynamic images showing cardiac tamponade (Figure 1).

**Figure 2:**
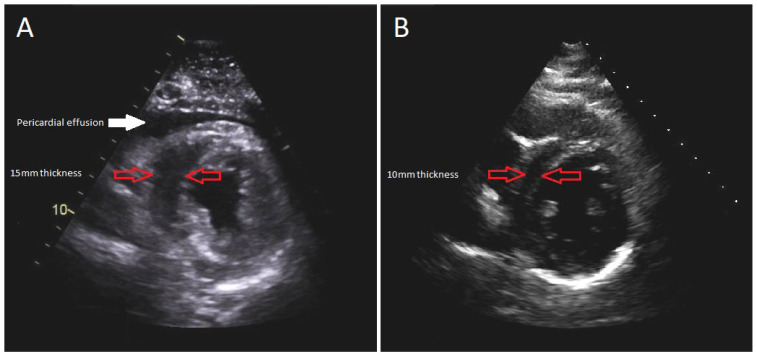
Parasternal short-axis view of TTE: (**A**) during the acute illness revealing 15 mm LV wall thickness; (**B**) 1-month follow-up demonstrating 10 mm LV wall thickness.

The patient was intubated, and an emergent subxiphoid pericardial window was performed with drainage of 250 milliliters of clear pericardial fluid. The patient’s hemodynamic parameters immediately improved, but not long after the patient became hypotensive and required a large volume of IV fluids totaling 14 L of crystalloid saline. Norepinephrine infusion was also initiated at the rate of 0.1 micrograms/kilograms/minute. The patient’s troponins continued to increase with repeat TTE, demonstrating decreased left ventricular (LV) systolic function to 44% along with moderately increased concentric LV wall thickness (Figure 2A). Despite receiving significant amounts of crystalloid fluid and continuous norepinephrine of 0.15 micrograms/kilograms/minute, she remained in refractory shock, at which time epinephrine was added at the rate of 0.07 micrograms/kilograms/minute.

The sepsis workup included serial blood cultures, urine cultures, and pan-CT imaging, which failed to point towards any source of infection. Cultures from pericardial fluid and tissue, including mycobacterial and fungal cultures, remained negative.

**Figure 3:**
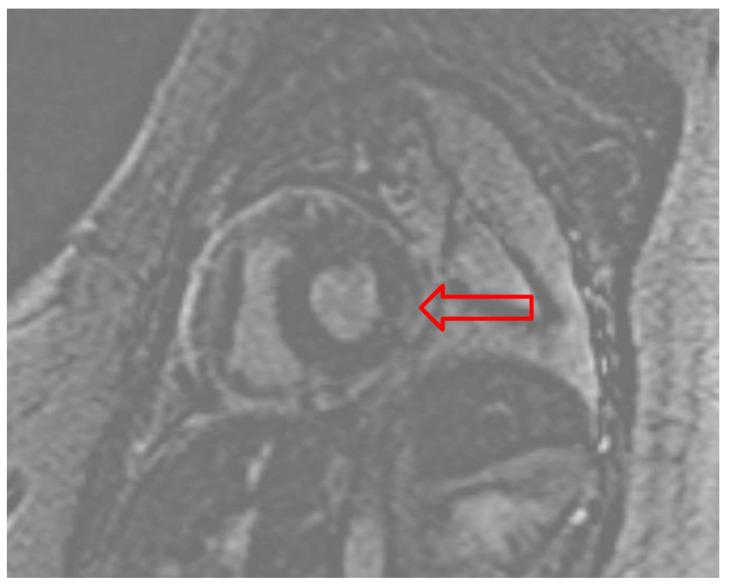
Gadolinium enhanced cardiac MRI pointing to the patchy sub-epicardial delayed enhancement of the basal lateral/inferolateral wall (red arrow).

Due to suspicion of fulminant myocarditis causing cardiogenic shock, the patient underwent RV endomyocardial biopsy (EMB). A 7.5 French bioptome was inserted into the sheath with five RV biopsy specimens obtained. Histopathology results were negative for myocarditis (evaluated with CD3, CD20, and CD68 immunohistochemical staining), but cardiac magnetic resonance imaging (cMRI) reported diffuse patchy myocardial edema along the inferolateral LV wall with minimal patchy sub-epicardial delayed enhancement consistent with myocarditis (Figure 3).

After one week of intensive care management with volume resuscitation and inotropic/vasopressor support, the patient was able to maintain hemodynamic stability. She was significantly hypervolemic, with notable anasarca. For the following 2 weeks, she was gradually diuresed, and by the time of discharge, she was net-negative 12 L. Her pericardial drain was removed 10 days after the pericardial window procedure.

Her hospital course was complicated by rhabdomyolysis, acute kidney injury, and intensive care unit-acquired weakness requiring intensive physical therapy. After 20 days of hospitalization, she was discharged to a rehabilitation facility. Her discharge medication regimen consisted of colchicine 0.6 milligrams daily for an anticipated 3-month course and oral furosemide 40 milligrams daily. After one month, a follow-up TTE demonstrated recovered systolic function with LV ejection fraction of 62%, resolution of myocardial edema, and no evidence of recurrent pericardial effusion (Figure 2B). No long-term complications were encountered on a subsequent follow-up.

## 3. Discussion

Patients with influenza infection most commonly present with non-specific viral-prodrome symptoms followed by a spectrum of respiratory illness [7]. Cardiovascular symptoms are often mild and under-recognized, particularly in young and healthy individuals; however, they can be fulminant and manifest as cardiogenic shock and/or obstructive shock due to cardiac tamponade [1,8]. Previous studies suggest a significant proportion of patients with influenza infection have unrecognized, “silent” myocardial injury, mainly manifested by elevated cardiac biomarkers [9]. Although the association between influenza and cardiac sequelae has been documented, mechanisms beyond a general inflammatory response remain uncertain [1]. Direct effect (i.e., viral tropism) on the myocardium and pericardium has been studied, but an exacerbation of the existing cardiovascular disease, such as heart failure or coronary artery disease, remains the most prevalent cause of morbidity and mortality [7]. Therefore, influenza vaccination is recommended by cardiology societies for patients with preexisting cardiac comorbidities [10]. Fulminant myocarditis usually has a rapid onset, and patients either recover with complete functional and histologic resolution or expire due to profound heart failure or tamponade within a few weeks following the development of initial symptoms [11]. The non-specific and dramatic presentation commonly makes the diagnosis challenging [1].

Our patient had a typical presentation with viral prodrome symptoms preceding respiratory symptoms that quickly progressed to hemodynamic instability initially due to tamponade but ultimately due to cardiogenic and distributive shock. Point of care ultrasound (POCUS) is a valuable tool for establishing the diagnosis in hemodynamically unstable patients, as it can rapidly differentiate the type of shock [12]. Formal TTE is also often necessary to evaluate ventricular function and pericardial morphology [13,14]. In our patient, LV dysfunction with increased concentric wall thickness resembling myocardial hypertrophy was an unusual finding in a young individual without prior cardiac history or hypertension. The etiology was suspected to be myocardial edema due to acute influenza infection, which was later confirmed on cMRI. Despite the cMRI revealing minimal patchy sub-epicardial delayed enhancement, the RV EMB was negative for myocarditis. False negative biopsy results, however, are not uncommon, mainly due to sampling error [15]. The sub-epicardial location of the inflammation also likely contributed to the negative RV EMB.

A literature review of transient ventricular wall thickening associated with influenza myocarditis or myopericarditis reveals a handful of cases diagnosed on cardiac imaging [6,16,17,18,19,20] or autopsy [21]. Reversal of wall thickening was observed in all recovered cases within 2 weeks. A study of 25 patients with acute lymphocytic and eosinophilic myocarditis conducted by Hiramitsu et al., demonstrated an association between ventricular wall thickening and interstitial edema in fulminant myocarditis, followed by a near resolution of wall thickness 1–2 weeks after the disease onset [22]. Furthermore, the sudden increase in LV wall thickness due to acute myocarditis was shown to have an inverse correlation with stroke volume, causing not only systolic dysfunction but also reduced diastolic filling [23].

Supportive management remains the cornerstone of influenza myocarditis treatment [1]. In fulminant cases with severe ventricular dysfunction, patients may require inotropic and/or vasopressive support and occasionally short-term mechanical circulatory support (MCS; e.g., intra-aortic balloon pump, extracorporeal membrane oxygenation (ECMO), or ventricular assist devices), which may not be readily available; early recognition and transfer to an ECMO-capable facility when necessary can be life-saving [1]. Conversely, isolated cardiac tamponade may be treated with pericardiocentesis, usually with prompt resolution of symptoms and restoration of normal hemodynamics [13].

Traditional antiviral medications, such as neuraminidase inhibitors (NAI; oseltamivir or zanamivir) are widely used for outpatient and inpatient influenza management [24]. To date, there is no study investigating the efficacy and the role of antiviral medications in the management of cardiac complications, although these medications are known to reduce symptom duration and progression to severe illness if used early in the clinical course (usually within 48 h) [1,25]. Recently, cases of successfully treated influenza myocarditis with the newer antiviral medications, such as peramivir, have been reported [26,27]. Anti-inflammatory medications, including non-steroidal anti-inflammatory drugs (NSAIDs) and colchicine, are recommended by cardiology societies’ guidelines for initial bouts of acute pericarditis [13]. Alternatively, if NSAIDs or colchicine are contraindicated, glucocorticoids may be used, although there was a concern of increased recurrence rate compared to the other anti-inflammatory agents [28]. Further studies have shown a higher recurrence rate with higher doses of corticosteroid therapy, while low to moderate dosages were associated with a lower recurrence rate [28,29]. Our patient required vasoactive and inotropic support along with significant amounts of crystalloid saline infusions due to volume depletion and distributive shock. Standard medical management consisted of an antiviral agent (oseltamivir), colchicine, and a short course of corticosteroids. Although it remained unclear if antiviral therapy was helpful given its initiation beyond the recommended 48 h after symptom onset, hospitalized patients often receive NAI later following symptom onset to reduce the risk of complications [20,30]. Throughout the hospital course, our patient was evaluated for MCS, but eventually, she improved with conservative medical management.

Although prognosis is favorable, previous studies reported high mortality in hospitalized patients with influenza-related cardiac complications, ranging from 24% to 35%, mainly due to fulminant myocarditis, profound ventricular dysfunction, or sudden cardiac death [31,32,33]. There was a notably higher incidence of myocarditis cases during and after the 2009 H1N1 pandemic and is suspected to be the reason for unexplained deaths among young individuals with influenza given the typical histopathologic finding of acute myocarditis being reported in 30 to 50% of autopsy cases [34,35,36]. Similarly, increased mortality due to cardiac-related complications was noted during the other major pandemics of the 20th century: H1N1 (Spanish flu; 1918), H2N2 (Asian flu; 1957), and H3N2 (Hong Kong flu; 1968) [37] and, most recently, SARS-CoV-2.

If recognized early, fulminant myocarditis has a relatively good prognosis with aggressive management and circulatory support [1]. Patients with fulminant (viral) myocarditis who survive usually recover within a few weeks, with an excellent long-term outcome and estimated 1- and 11-year survival rates of 95% and 93%, respectively [38]. Long-term complications are infrequently encountered [1,3].

## 4. Conclusions

Influenza myocarditis is usually a mild and self-limited illness, although fulminant forms are sporadically encountered. Cardiac tamponade and transient ventricular wall thickening due to myocardial edema leading to cardiogenic shock are rare but life-threatening complications that should be considered in patients of any age with chest pain and hemodynamic instability following a viral infection. A high clinical suspicion is needed for prompt recognition and timely management to avoid high mortality. Furthermore, vigorous supportive management is crucial for a positive outcome. Patients who recover usually have a complete functional and structural (histologic) resolution.

## Figures and Tables

**Table 1:** Daily vital signs, hemodynamic parameters, pertinent laboratory findings, and treatment timeline.

Day	0	1	2	3	4	5	6	7	8	9	10	11	12	13	14	19 Discharge	1 Month Follow Up
** Temp (°C) **	35.8	35.6	39.9	38.2	37.6	39.0	37.4	38.0	38.0	36.8	36.7	37.0	36.9	37.2	37.0	36.9	36.4
**HR (beats/min)**	146	136	135	97	101	111	122	107	102	106	104	111	106	111	105	85	86
**BP (mmHg)**	91/71	94/78	87/58	82/53	100/82	104/70	109/65	100/59	108/61	115/65	113/59	115/66	121/69	110/62	133/72	124/78	110/76
**Intake (ml)**		6000	8000	4000													
**Output (ml)**		450	550														
**Weight (kg)**	92.8	96.6	97.7		118	118	109	110	110						94.3	92.4	88.1
**LVEF (%)**	57	56	44	43					63								62
																	
**Hgb (g/dL)**	21.3	19.1	17.5	10.8	12.6	9.9	10.3	9.6	8.8	9.2	9.4	9.4	9.1	8.4	8.1	8.5	12.8
**Hct (%)**	64.3	57.4	54.2	32.2	38.0	31.5	32.7	31.8	29.0	29.7	30.1	30.1	29.1	26.8	25.8	26.9	40.7
**Plt (10^3^/mcL)**	415	310	207	96	97	131	147	200	223	208	226	86	124	129	133	167	284
**WBC (10^3^/mcL)**	16.7	25.5	31.0	21.5	28.1	21.7	27.4	24.2	20.6	16.5	17.4	15.9	16.2	15.8	13.3	7.5	9.0
**Cr (mg/dL)**	0.96	0.71	1.09	0.7	0.8	1.0	0.9	1.1	1.8	2.1	2.2	2.1	1.8	2.0	1.85	2.0	1.2
**CK (U/L)**	1181	1831	2397	8397	17,786	15,636	13,226	22,990	18,793	13,744	10,029	5985					
**Trop-T (ng/L)**	0.06	0.106	0.77	0.46	0.38												
**CK-MB (ng/mL)**	10.8	18.6	29.4														
**Pro-BNP (pg/mL)**	1208																
**Lactate (mmol/L)**	2.8	4.6	6.8	6.2	4.7	2.7	2.1										
																	
	**Methylprednisolone 40 mg every 6 h**																
	**Colchicine 0.6 mg two times daily**															**Colchicine 0.6 mg daily**	
**Management:**										**IV Lasix diuresis**						**Lasix 40 mg daily**	
			**Norepinephrine at 0.1 mcg/kg/minute**														
				**Epinephrine at 0.07 mcg/kg/minute**													
	**Oseltamivir 75mg two times daily**																
		**Vancomycin + piperacillin-tazobactam**															
**Interventions:**		**Subxiphoid pericardial window**	**RV endomyocardial biopsy**										**Pericardial drain** **removed**				

Legend: HR—heart rate; BP—blood pressure; PA—pulmonary artery; LVEF—left ventricular ejection fraction; Hgb—hemoglobin; Hct—hematocrit; Plt—platelet count; WBC—white blood cell count; Cr—creatinine; CK—creatine-kinase; Trop T—troponin T; CK-MB—creatine-kinase-MB; Pro-BNP—proB-type Natriuretic Peptide.

## Data Availability

Not applicable.
